# Physiology of Women

**Published:** 1851-09

**Authors:** 


*Physiology of Women.*—The difference that exists between the European and the Hindoo must be sought *in*

*race.* When it is recollected that the consummation of marriage among the Hindoos has taken place, at the latest, on the arrival of puberty, during the lapse of more than 3,000 years, and that the practice is sanctioned by ancient laws, and consecrated by custom, it is easy to conceive that those females who were the latest in reaching puberty would be the least sought after for wives; that such women would not unlikely, in many instances, remain unmarried; and that thus (owing to the origination of a preference on this ground in the selection of wives, operating through a long period of time) Hindoo women would gradually come to consist, in a proportion different from that in Europe or elsewhere, of such as by constitution are early nubile. To me there seems nothing extravagant or far fetched in this supposition. The production of a like state of things in England, in any particular district, is quite conceivable. Nothing is better established than that early puberty is a family peculiarity. Let us, then, only suppose families, possessing this kind of constitution, to intermarry, and the peculiarity in question would be propagated, extended, and transmitted, and so a race distinguished by it would be produced.

Dr. Goodeve, in his reply to queries, states that he has known menstruation in the Hindoo continue till the age of fifty. In the table for Toomkoor, the mean age is forty-five years nearly. There are also thirteen instances, headed "menstruation late in life," in the Baboo's table. Six of these were persons still menstruating, one 50, and one at each of the following ages: 56, 63, 64, 67, and 68; likewise, seven that had ceased, at the following ages: 56, 57, 58, 59, 60, 65, and 80 years —instances, of course, as late could be paralleled in any country. No great weight, however, is to be attached to exceptional cases. If we may judge from the Toomkoor table, the mean age at the cessation of the catamenia is full two years earlier than it is here.

The age for a first birth is considerably later in the Deckan than it is in Calcutta. At Bombay, the mean age is little short of seventeen years; in Bangalore, sixteen years and five months; while in Calcutta, the mean is fourteen years and eight months. But, taking the mean age for India, which is fifteen years six and a half months, we still have a social phenomenon of a very surprising kind, when compared with the age for the same event in Europe. From a register of the time of life at a first parturition in five hundred women of Manchester, chiefly of the class of manufacturing laborers, I find the mean age to be twenty-three years, whereas the mean age for a first birth in Hindostan is earlier by nearly eight years! Moreover, in the English register there is no instance of parturition under the age of 17, while in the Hindoo table, one-half occurs where the mother is under 16, and not one in ten is of the age of twenty and upwards.

A fact, hitherto, I apprehend, unknown to Europeans, is the law of the Shastras, that females should be given in marriage *before* the occurrence of menstruation; and that, should consummation not take place until after this event, the marriage is a sin. Accordingly, it is the custom, in Lower Bengal, to send the girl at the age of nine years to the house of her husband, unless the latter be so distant that it cannot be done: and two ancient Hindoo sages are of the opinion that if the marriage is not consummated before the first appearance of the catamenia, the girl becomes "degraded in rank." Testimony to the same effect is given by Professor Webb, who likewise joins the Baboo in thinking that, in those alleged instances of very early menstruation, a discharge of blood from injury in premature intercourse is the real explanation of the occurrence; a supposition worthy of consideration, when endeavoring correctly to estimate the value of the Calcutta tables. At Bangalore, it would seem that this revolting custom of consummating the marriage before puberty does not obtain, the husband refraining from taking his wife to his own house till not less than sixteen days have elapsed subsequently to puberty. Customs, we know, differ, in different parts of our great Eastern Empire. I had occasion formerly to refer to the diversity of manners, and even morals, in so vast a country. In the present instance it might readily escape notice, when speaking of Calcutta and Bangalore, that these two cities are about as widely apart as to distance, and probably not less diverse in customs and manners, than are Copenhagen and Madrid, the capitals of Denmark and Spain.

Viewing, as we must do, with pity and disgust, the infantile marriages of Hindostan, it is some consolation to learn from Professor Webb that the more intelligent Hindoos of the present day hesitate not to pronounce these early unions as the monster evils of their coutry. —*Roberton on Physiol. and Dis. of Women.*

